# Dimethyl fumarate prevents acute lung injury related cognitive impairment potentially via reducing inflammation

**DOI:** 10.1186/s13019-021-01705-6

**Published:** 2021-11-12

**Authors:** Xiaowei Wang, Yanbo Wang, Haiyan Pan, Ci Yan

**Affiliations:** 1 Department of Respiratory, The Third Affiliated Hospital of Zhejiang Chinese Medicine University, Hangzhou City, 310000 Zhejiang Province China; 2 Department of Neurology, The Third Affiliated Hospital of Zhejiang Chinese Medicine University, Hangzhou City, 310000 Zhejiang Province China; 3 Department of Endocrinology, The Third Affiliated Hospital of Zhejiang Chinese Medicine University, Hangzhou, 310000 China; 4 Departments of Psychiatry, Affiliated Mental Health Center, Zhejiang University School of Medicine, No. 305 Tianmu Shan Road, Hangzhou City, 310000 Zhejiang Province China

**Keywords:** Acute lung injury, Dimethyl fumarate, Inflammation, Cognitive deficits

## Abstract

**Objective:**

Dimethyl fumarate (DMF) has been reported to exert a protective role against diverse lung diseases and cognitive impairment-related diseases. Thus this study aimed to investigate its role on acute lung injury (ALI) and related cognitive impairment in animal model.

**Methods:**

C57BL/6 mice were divided into four groups: control group, DMF group, ALI group, and ALI + DMF group. For ALI group, the ALI mice model was created by airway injection of LPS (50 μL, 1 μg/μL); for ALI + DMF group, DMF (dissolved in 0.08% methylcellulose) was treated twice a day for 2 days, and on the third day, mice were injected with LPS for ALI modeling. Mice pre-administered with methylcellulose or DMF without LPS injection (PBS instead) were used as the control group and DMF group, respectively. Morris water maze test was performed before any treatment (0 h) and 6 h after LPS-induction (54 h) to evaluate the cognitive impairment of mice. Next, the brain edema and blood brain barrier (BBB) permeability of ALI mice were assessed by brain water content, Evans blue extravasation and FITC-Dextran uptake assays. In addition, the effect of DMF on the numbers of total cells and neutrophils, protein content in BALF were quantified; the inflammatory factors in BALF, serum, and brain tissues were examined by ELISA, qRT-PCR, and Western blot assays. The effect of DMF on the cognitive impairment-related factor HIF-1α level in lung and brain tissues was also examined by Western blot.

**Results:**

DMF reduced the numbers of total cells, neutrophils and protein content in BALF of ALI mice, inhibited the levels of IL-6, TNF-α and IL-1β in BALF, serum and brain tissues of ALI mice. The protein expressions of p-NF-κB/NF-κB and p-IKBα/IKBα was also suppressed by DMF in ALI mice. Morris water maze test showed that DMF alleviated the cognitive impairment in ALI mice by reducing the escape latency and path length. Moreover, DMF lessened the BBB permeability by decreasing cerebral water content, Evans blue extravasation and FITC-Dextran uptake in ALI mice. The HIF-1α levels in lung and brain tissues of ALI mice were also lessened by DMF.

**Conclusion:**

In conclusion, DME had the ability to alleviate the lung injury and cerebral cognitive impairment in ALI model mice. This protective effect partly associated with the suppression of inflammation by DMF.

## Introduction

Acute lung injury (ALI) and its more serious form of respiratory distress syndrome (ARDS) is reported with a widely variable incidence while can lead to death in ALI patients [1]. Pulmonary infection, srious pneumonia, trauma, shock, sepsis, cardiothoracic surgery and other related factors are contributed to the occurrence of ALI [2, 3]. In perioperative period of cardiothoracic surgery, the surgeon needs to maintain a suspicion for the risk of ALI, for which is correlated with the surgical prognosis and the causes of operative death [4, 5]. ALI is characterized by rapidly acting pulmonary edema, hypoxemia, accumulation of activated inflammatory cells, mass migration of neutrophils, and inflammatory processes [6, 7]. Therefore, the inflammatory response and hypoxia are critical in ALI. Interestingly, studies have confirmed that patients with ALI/ARDS have neurocognitive impairment, which seriously affects the patient’s life quality [8, 9]. Recently, it has been manifested that “two-hit”-triggered ALI might impair the cognitive function in mice through excessive inflammation, leading to the destruction of the blood–brain barrier (BBB) and thus impair cognitive function [10]. Although the current recognizing of the nosogenesis and elements affecting the prognosis of patients has improved, there is still a lack of effective drugs for the treatment of ALI/ARDS [11]. Therefore, it is necessary to find new drugs with high efficiency and low toxicity, which is of great significance for the treatment of ALI.

Dimethyl fumarate (DMF) is an agonist of nuclear factor (erythroid-derived 2)-like 2 (Nrf2), the rapid and durable ability of DMF has been confirmed in clinical trials in patients with multiple sclerosis [12, 13]. DMF also has been reported to exert a pivotal role in the pathogenesis of lung diseases, such as pulmonary arterial hypertension and lung fibrosis, lung carcinogenesis, asthma [14, 15]. Related studies showed that the protective effect against lung diseases were partly owing to anti- inflammation and oxidative stress ability. However, the role of DMF in ALI has not been cleared yet. More over, clinical study showed that DMF was associated with the slowing cognitive impairment and significant improvements in quality of life and psychosocial function in multiple sclerosis patient [16].

Lipopolysaccharide (LPS) is the major component of the cell wall of Gram-negative bacilli and is often utilized to induce the experimental ALI model, LPS infection can simulate the clinical ALI caused by Gram-negative bacillus infection (such as various sepsis) and septic shock [17]. One study has manifested that the acute inflammatory response induced by LPS could notably promote the presence and development of ALI [18]. In this study, we aimed to establish a mouse model of LPS-triggered ALI and evaluate the protective effect of DMF on LPS-triggered ALI in mice.

## Materials and methods

### Animals

A total of 40 SPF-grade healthy male C57BL/6 mice (6–8 weeks old, weight 22 ± 2 g) were obtained from Shanghai SLAC Laboratory Animal Co., Ltd. (Shanghai, China). All mice are kept in standard squirrel cages with a light/dark cycle of 12/12 h (h), a relative humidity of 45 to 50%, and an ambient temperature of 22 to 23 °C. All animals were adaptively fed for one week and had free access to food and water. Following the principles of Guide for the Care and Use of Laboratory Animals, we tried our best to reduce the pain of mice during the experiment, the experiments were allowed by the Committee of Laboratory Animals of Hangzhou Eyong Biotechnological Co., Ltd. Animal Experiment Center (Hangzhou, China).

### Pre-treatment and establishment of ALI mouse model

All mice were randomly separated into four groups (n = 10 per group): control group, DMF group, ALI group, and ALI + DMF group. In the DMF group and ALI + DMF group, mice were given a dose of 25 mg/kg DMF (242926, Sigma-Aldrich, USA; dissolved in 0.08% methylcellulose) by gavage twice a day for 2 days (during 0 to 48 h) [19]. In the control group and ALI group, mice were subjected to the same amount of methylcellulose as a vehicle.

**Fig. 1:**
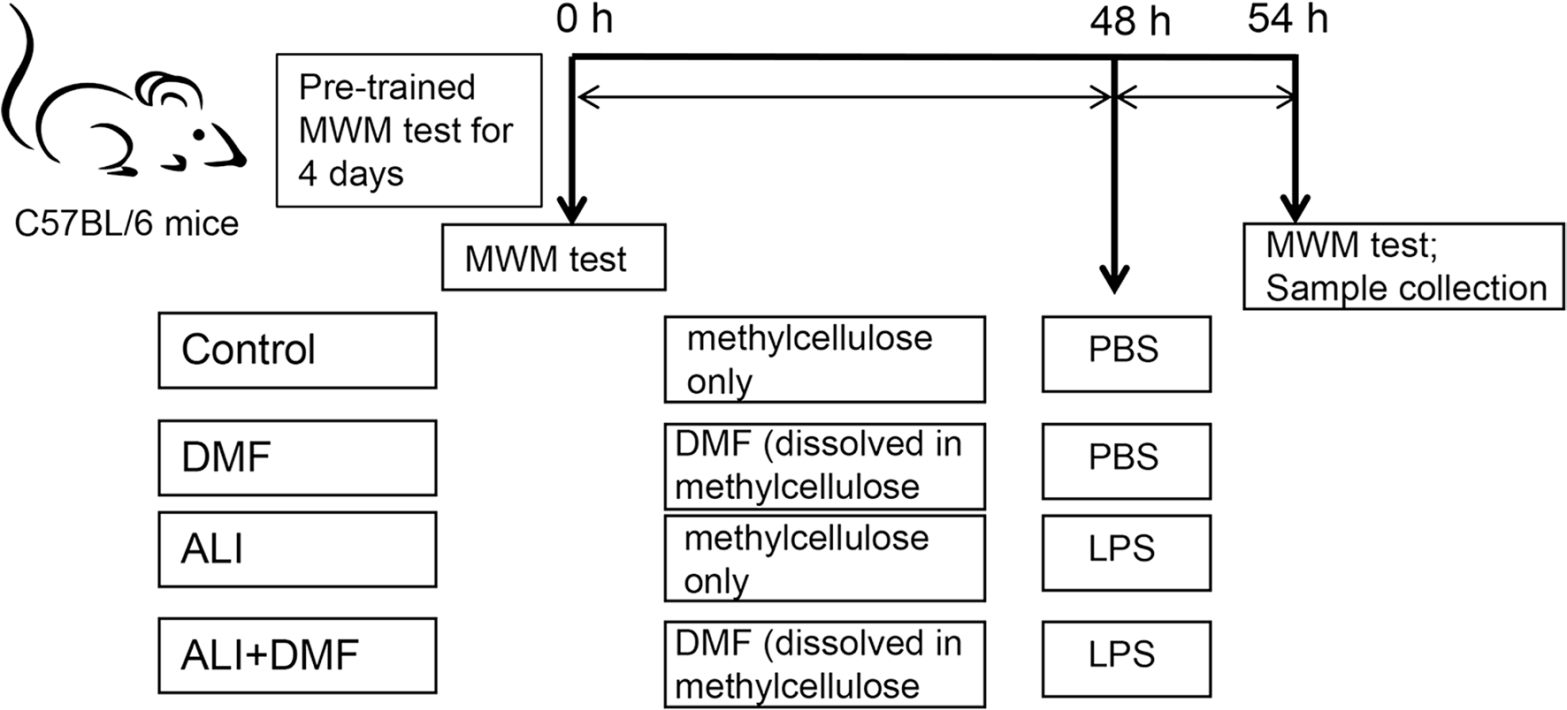
Schematic representation of experimental design. C57BL/6 mice were divided into four groups: Control group, DMF group, ALI group, and ALI + DMF group. Before the therapeutic experiment, mice in each group were pre-trained with Morris water maze (MMM) test for 4 consecutive days. Afterwards, during the experiment, the DMF group and ALI + DMF group mice were given a dose of 25 mg/kg DMF by gavage twice a day for 2 days (during 0 to 48 h), the control group and ALI group mice were subjected to methylcellulose as a vehicle. At 48 h, ALI and ALI + DMF group mice were airway injected with LPS, while Control and DMF group mice were administered with PBS buffer. At 0 h and 54 h, mice in each group were tested with Morris water maze test. After that, all mice were sacrificed and samples were collected

After 2 days of DMF pre-treatment, ALI model was subsequently induced in ALI and ALI + DMF group mice. As previously described by Tang et al. [20], the mice were anesthetized by inhaling isoflurane (792632, Sigma-Aldrich, USA) at a dose of 100 mg/kg. After the mouse was fixed on a small plate to keep still, the mouth was opened with the help of wireless visual mirror and keep under the light source. After further opening the glottis, the vein cannula was grasped with our right hand and quickly inserted it into the glottis until it reached the airway. Thereafter, a pre-prepared solution of 50 μL LPS (1 μg/μL, L2630, Sigma-Aldrich, USA) was injected into the airway through the cannula. After administration, the mice were gently shaken and gently tapped on the chest to ensure that LPS was evenly distributed between the left and right lungs. The control and DMF group mice were administered with PBS buffer of the same volume according to the same method. Figure 1 was the schematic representation of the experimental design.

### Morris water maze (MWM) test

MWM test was performed before DMF pre-treatment (at 0 h) and 6 h after LPS administration (at 54 h) to evaluate the spatial learning and reference memory periodically in mice with or without ALI according to the Ref. [21]. Briefly, the water maze was mainly composed of a barrel with a diameter of 150 cm and a height of 60 cm and an automatic video analysis system. During the positioning navigation experiment, the training phase lasted for 4 consecutive days, 4 times a day. They were placed on an underwater platform to adapt for 30 s before entering the water. We recorded the swimming distance and time of the mice from the different entry points in four quadrants to the platform within 60 s and took the average score to enter the final statistics. After the positioning navigation experiment, the platform was removed on the next day. Mice were placed into the water facing the pool wall from the entry point of the quadrant on the opposite side of the platform. The swimming track of mice was recorded, the escape latency and path length were also measured. Longer escape latency time and path length reflect the existence of the cognitive impairment.

### Evans blue extravasation

BBB permeability was evaluated by measuring the Evans blue extravasation [22]. Evans blue dye can bind to serum albumin, a major protein in the blood, while in a brain where the BBB is destructed, the Evans blue-bound albumin will pass from the bloodstream into brain tissue, so the Evans blue extravasation indicates the status of BBB. After the second MWM test, half of the mice in each group were injected with 2% (w/v) Evans blue dye saline solution (4 mL/kg, IE0280, Solarbio, China) intravenously. Two hours later, the hearts of mice were perfused with PBS under deep anesthesia. Next, the mouse brain was removed, the hippocampus was isolated, weighed. They were then shredded before homogenization with *N*-dimethylformamide (227056, Sigma-Aldrich, USA). The harvested homogenates were placed at room temperature away from the light for 3 days and then centrifuged (10,000 g × 25 min). After that, the supernatant was examined through a fluorescence spectrometry (excitation wavelength was 620 nm and emission wavelength was 680 nm). The total EB content in the brain was quantified as ng/mg brain tissue using Evans blue-albumin as standard.

### FITC-Dextran extravasation measurements

BBB permeability was evaluated by measuring the FITC-Dextran extravasation [23]. After the second MWM test, another half of the mice were intravenously injected with 1 mL of 0.5 mg/mL FITC-Dextran saline solution (46945, Sigma-Aldrich, USA). Two hours later, the mice were irrigated with 0.1 M PBS (pH 7.4). We quickly removed mouse brain tissue and separated the hippocampus, and then homogenized in 0.1 M PBS (pH 7.4) containing 20% (w/v) trichloroacetic acid solution (T6399, Sigma-Aldrich, USA). After centrifugation, the fluorescence spectrometry (excitation wavelength was 493 nm and emission wavelength was 520 nm) was sued to assess the fluorescence intensity.

### Sample collection

After the deep anesthesia, the blood samples from the heart were extracted and centrifuged to obtain serum samples, which was then stored at − 80 °C for subsequent cytokine determination. The the median sternum of the mouse was cut to expose the lungs. After ligation of the right hilum, we performed left lung lavage to obtain the broncho-alveolar lavage fluid (BALF). After that, the lung tissues were rapidly removed and quickly frozen at − 80 °C for subsequent experiments. A part of the brain tissue was used to assess brain water content, another part of the brain tissue was quickly frozen at − 80 °C for subsequent experiments.

### Brain water content

The brain tissues were washed with PBS and then weighed (wet weight). Afterwards, the brain tissues were placed in a 65 °C oven for 48 h to obtain “dry” weight. The water content was counted as follows: water content (%) = (wet weight − dry weight)/wet weight × 100%.

### Analysis of total cells and neutrophils in BALF

The collected BALF was centrifuged at 4 °C at 1500 r/minutes (min) for 10 min to obtain cell pellets. After the cell pellets were suspended in 1 mL PBS, a hemocytometer (Sigma-Aldrich, Merck KGaA) and Wright-Giemsa staining of cytospin preparations were used to determine the numbers of total cells and neutrophils in BALF.

### BALF total protein assay

The BALF supernatant was remoced from − 80 °C and mixed thoroughly. To detect the alveolar capillary damage and vascular leakage, we used Lowry Protein Assay Kit (PC0030, Solarbio, China) to calculate the total protein concentration of BALF supernatant [24].

### ELISA

The mouse IL-6 kit (MM-0163M1), mouse TNF-α kit (MM-0132M1), and IL-1β kit (MM-0040M1) were obtained from MEIMIAN (China). In brief, BALF supernatant and serum samples were added to the sample well, respectively, followed by the addition of IL-6, TNF-α or IL-1β kit. After reaction, a microplate reader (CMaxPlus, MD, USA) was used to assess the absorbance of each well at 450 nm.

### QRT-PCR

Total RNA of the brain tissues and serum was acquired using TriReagent (T9424, Sigma-Aldrich, USA). Next, cDNA was acquired using the reverse transcription kit (CW2569, CWBIO, China). QRT-PCR was utilized for examining the levels of IL-6, TNF-α, and IL-1β, which was carried out with the SYBR Green qPCR kit (CW2601, CWBIO, China) on a PCR instrument (Mastercycler, Eppendorf, Germany). GAPDH was served as the reference gene and data were expressed as 2^−ΔΔCt^ method. The sequences of the primers are listed 5′ to 3′: TNF-α, (F) TATGGCTCAGGGTCCAACTC; (R) CTCCCTTTGCAGAACTCAGG; IL-1β, (F) GACCTTCCAGGATGAGGACA; (R) AGGCCACAGGTATTTTGTCG; IL-6, (F) CCGGAGAGGAGACTTCACAG; (R) TCCACGATTTCCCAGAGAAC; GAPDH, (F) ATGACATCAAGAAGGTGGTG; (R) CATACCAGGAAATGAGCTTG.

### Western blot

Proteins in the brain tissues and lung tissues were harvested by a RIPA buffer (P0013D, Beyotime, China) and their concentrations were qualified with a BCA Kit (pc0020, Solarbio, China). After denaturation, the protein samples were separated by electrophoresis. Proteins in the gel were transferred to a nitrocellulose membrane (10600023, GE Healthcare Life, USA), which was then sealed a 5% skim-milk. After that, they were reacted with primary antibodies at 4 °C overnight. After washing, they were reacted with anti-rabbit HRP (1:5000, #7074, CST, USA) at 37 °C for 1 h. In the end, the protein signals were developed by the ECL reagent (35055, Pierce, USA) in a gel imaging system (A44114, Invitrogen, USA). The primary antibodies of TNF-α (ab205587, 1:1000), IL-1β (ab254360, 1:1000), p-NF-κB (ab76302, 1:1000), NF-κB (1:5000, ab32536), p-IKBα (1:10,000, ab133462), IKBα (1:5000, ab32518), HIF-1α (1:1000, ab179483), and GAPDH (1:5000, ab199554) were obtained from Abcam (UK).

### Statistical analysis

The statistical analysis was implemented with SPSS software (16.0, IBM, USA). One-way ANOVA followed SNK test was employed for comparison among multiple groups, Dunnett’s T3 test was employed for those with equal variances not assumed analysis. The data were described by mean ± standard deviation. *P* < 0.05 was designated as statistically significant.

## Results

### DMF alleviated ALI-associated lung injury

**Fig. 2:**
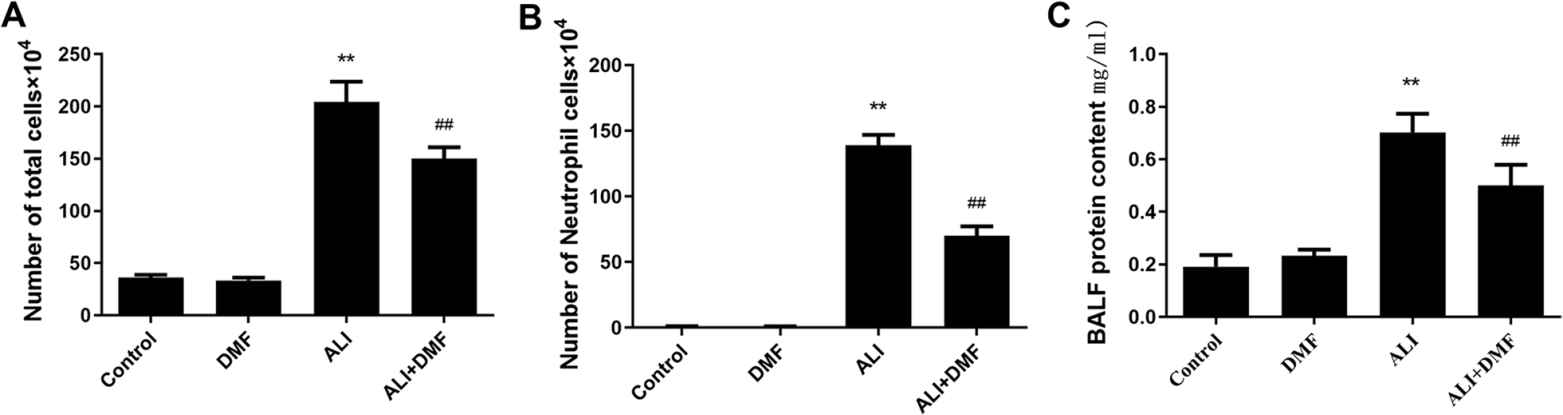
The effect of DMF on the numbers of total cells (**A**), neutrophils (**B**) and protein content (**C**) in the BALF of LPS-induced ALI model. The values are presented as means ± standard deviation (SD). *P < 0.05, **P < 0.01 versus control group; ^#^*P* < 0.05, ^##^*P* < 0.01 versus ALI group. *ALI* acute lung injury, *BALF* bronchoalveolar lavage fluid

As shown in Fig. 2A, B, DMF pretreatment alone did not obviously change the numbers of total cells and neutrophils in BALF compared to the control group, while LPS-induced ALI led to a sharp increase (*P* < 0.01). On the other hand, DMF administration caused a decrease of total cells and neutrophils in BALF (Fig. 2A, B, *P* < 0.01). Then, we evaluated the protein content in BALF. LPS treatment resulted in an evident increase of the protein content, while DMF pre- treatment partly inhibited the effect of LPS on protein content (Fig. 2C, *P* < 0.01).

### The role of DMF on the production of inflammatory cytokines in BALF and serum of LPS-triggered ALI mice

**Fig. 3:**
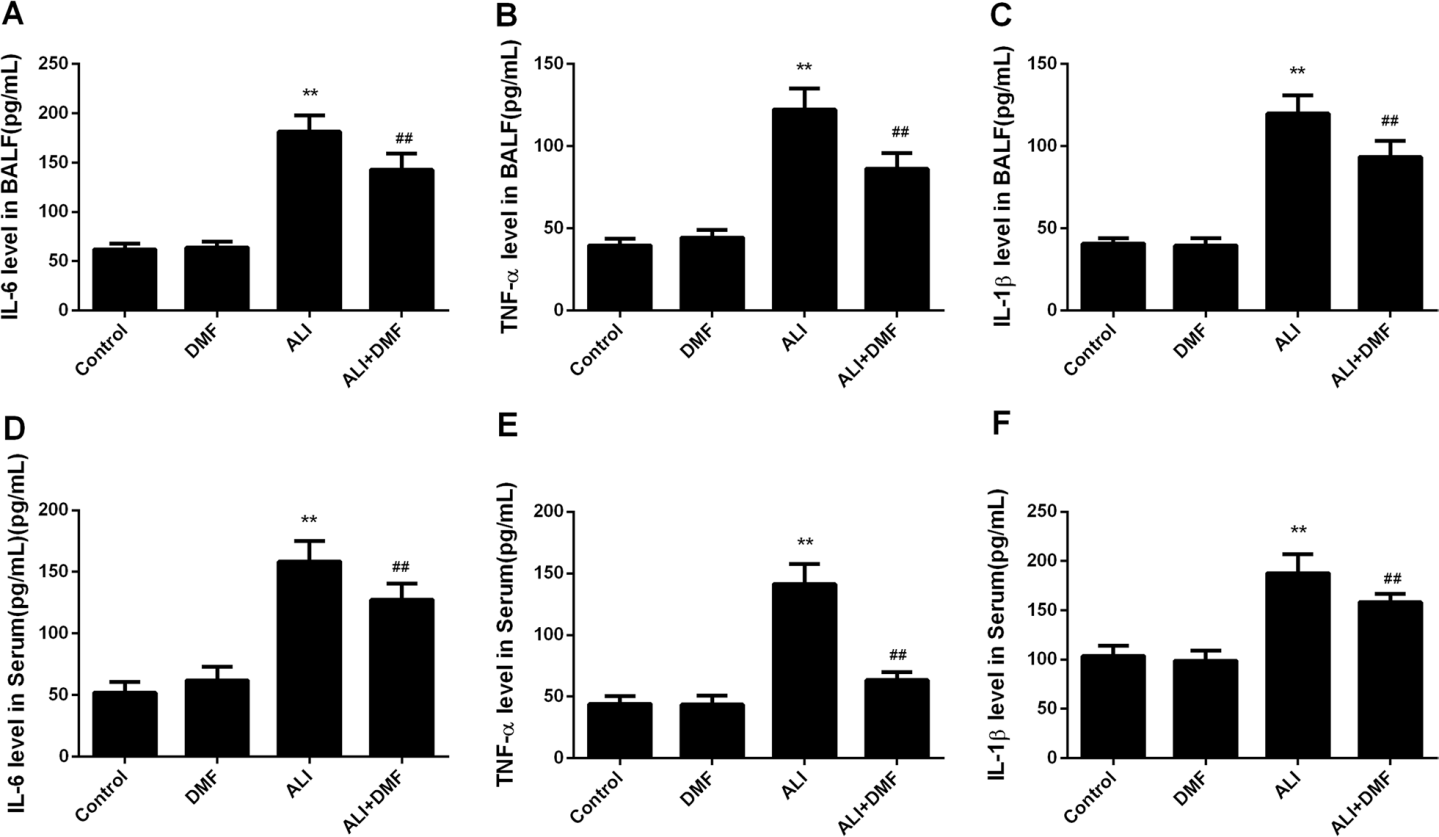
The effect of DMF on the production of inflammatory cytokines in BALF and serum of LPS-induced ALI model. The levels of IL-6 (**A**), TNF-α (**B**), IL-1β (**C**) in BALF and the levels of IL-6 (**D**), TNF-α (**E**) and IL-1β (**F**) in serum were measured using ELISA. The values are presented as means ± SD. **P < 0.01 versus control group; ^##^*P* < 0.01 versus ALI group

In Fig. 3A–F, our analysis of ALI-related inflammation-related markers in BALF and serum exhibited that LPS caused an increase in the contents of TNF-α, IL-1β and IL-6, while DMF pretreatment in ALI group partially offset the promotion of LPS (*P* < 0.01).

### The role of DMF on the inflammation-related markers level in serum and brain tissues of LPS-triggered ALI mice

**Fig. 4:**
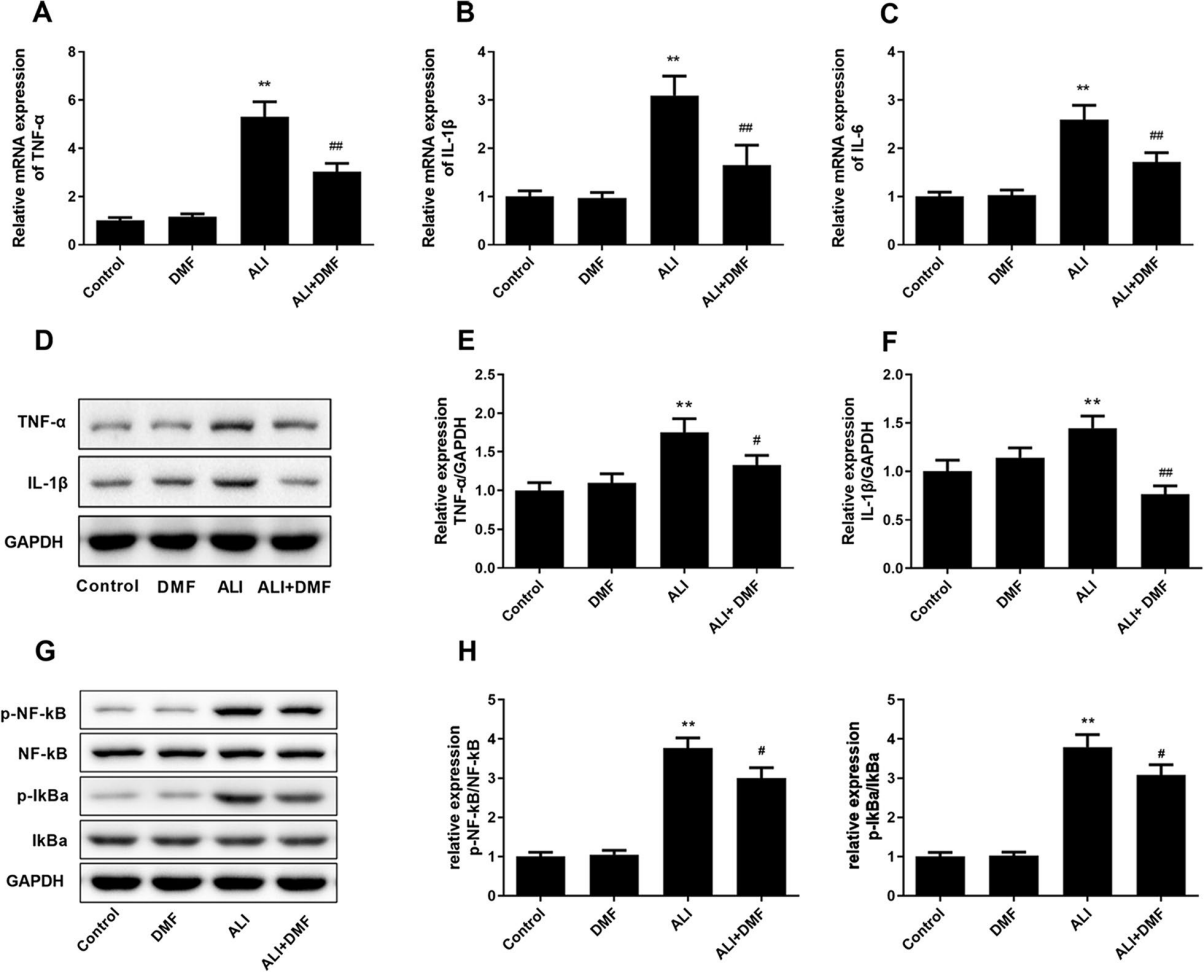
The effect of DMF on the expression of inflammatory cytokines in the serum and hippocampal tissues of LPS-induced ALI model. The mRNA expression of TNF-α (**A**), IL-1β (**B**) and IL-6 (**C**) were measured using qRT-PCR. The proteins expression levels of TNF-α and IL-1β were analyzed using the Western blot analysis (**D**). The proteins expression of TNF-α and IL-1β were quantified by compared with GAPDH (**E**, **F**). The proteins expression levels of p-NF-κB and p-IKBα were analyzed using the Western blot analysis (**G**). The ratios of p-NF-κB and p-IKBα were quantified by compared with GAPDH (**H**).The values are presented as means ± SD. **P < 0.01 versus control group; ^#^*P* < 0.05, ^##^*P* < 0.01 versus ALI group

We first detected the inflammatory cytokines in serum by qRT-PCR and confirmed that the TNF-α, IL-1β and IL-6 levels of serum in the ALI group were extremely enhanced than the control group (Fig. 4A–C, *P* < 0.01). Nevertheless, the elevated effect was inhibited by DMF pretreatment (Fig. 4A–C, *P* < 0.01). Meanwhile, we also tested the inflammatory cytokines in the brain tissues by Western blot, the same results were found in Fig. 4D–F (*P* < 0.05). Moreover, LPS caused an obvious raise in the ratios of p-NF-κB/NF-κB and p-IKBα/IKBα, which was partially reduced by DMF (Fig. 4G–H, *P* < 0.05).

### DMF alleviated the cognitive impairment in ALI mice

**Fig. 5:**
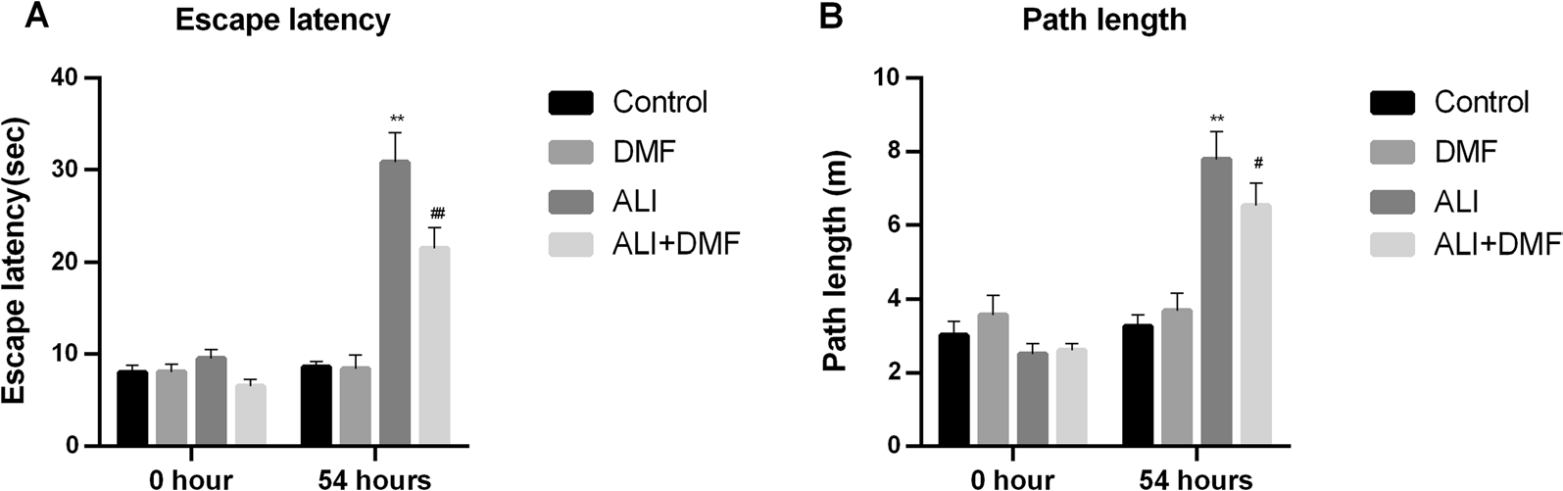
The effect of DMF on escape latencies (**A**) and path lengths (**B**) of LPS-induced ALI model in the Morris Water Maze. The values are presented as means ± SD. **P < 0.01 versus control group; ^#^*P* < 0.05, ^##^*P* < 0.01 versus ALI group

There was no evident difference in escape latency and path length of mice in each group at 0 h (Fig. 5A, B). After 54 h, LPS induced persistent cognitive impairment in mice, which was clearly reflected in escape latency and path length (Fig. 5A, B, *P* < 0.001). Interestingly, DMF pretreatment in ALI group partially offset the effect of LPS (Fig. 5A, B, *P* < 0.01).

### The role of DMF on the protection of BBB in LPS-triggered ALI mice

**Fig. 6:**
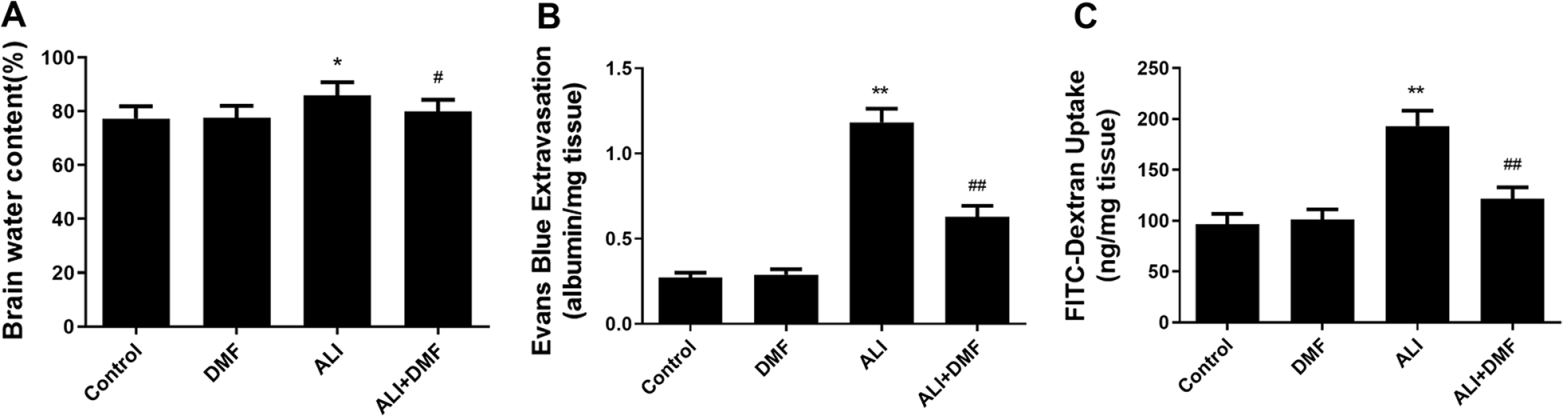
The effect of DMF on the protection of BBB in LPS-induced ALI model. Brain water content (**A**), Evans blue extravasation (**B**) and FITC-Dextran uptake (**C**) were assessed to evaluate BBB permeability. The values are presented as means ± SD. *P < 0.05, **P < 0.01 versus control group; ^#^*P* < 0.05, ^##^*P* < 0.01 versus ALI group

In order to check the permeability of BBB, we evaluated the brain water content, Evans blue extravasation and FITC-Dextran uptake in mice. DMF alone did not change the above mentioned effect (Fig. 6A–C). We discovered that LPS led to the increase of the brain water content, Evans blue extravasation and FITC-Dextran uptake (Fig. 6A–C, *P* < 0.05). Nevertheless, the enhanced effect was reversed by DMF pretreatment (Fig. 6A–C, *P* < 0.05).

### The effect of DMF on the HIF-1α level of lung and brain tissues in LPS-induced ALI mice

**Fig. 7:**
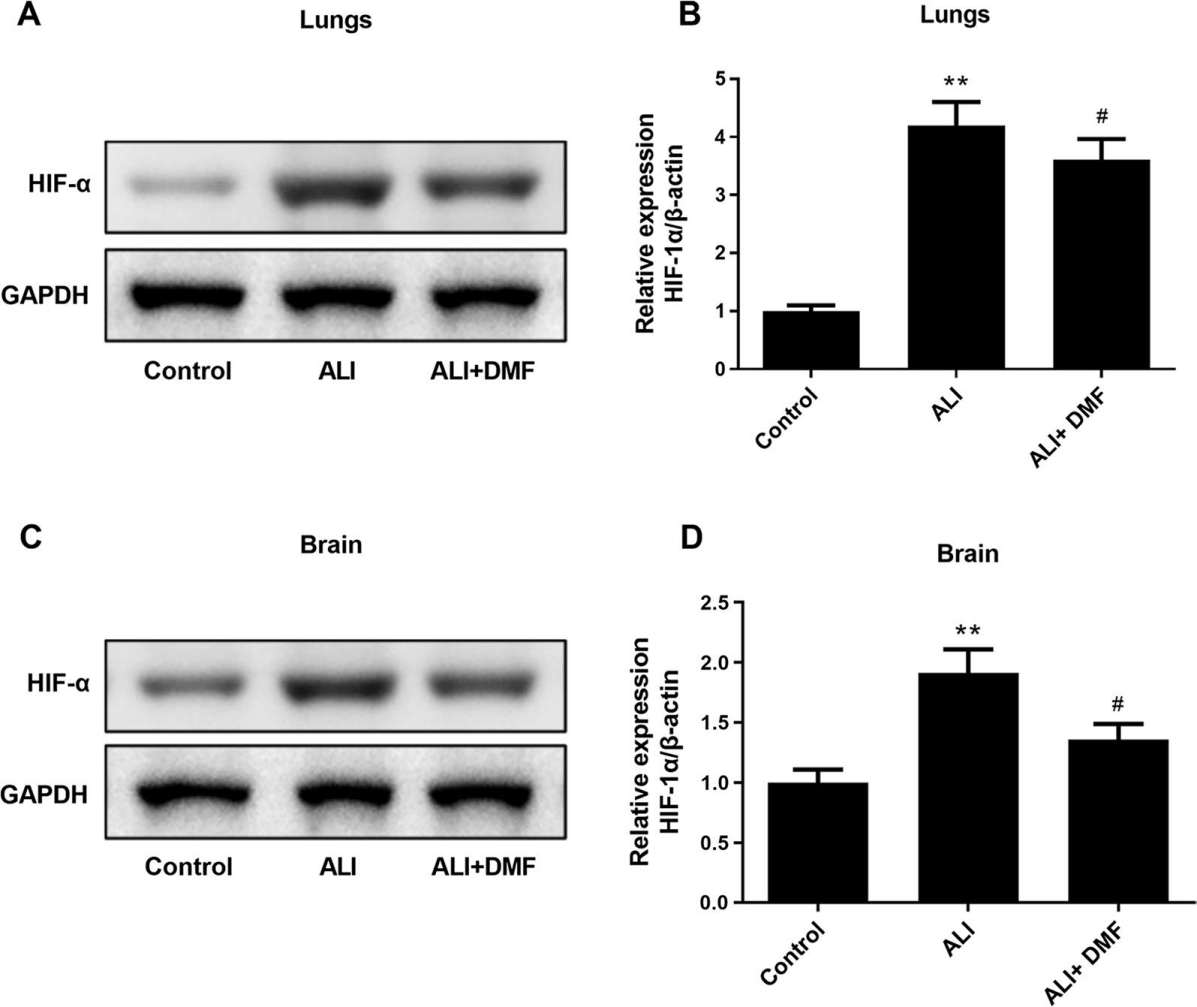
The effect of DMF on the role of hypoxia in cognitive impairment induced by LPS. Western blotting analysis was utilized to assess the expression of HIF-α in lung and brain (**A**, **C**). The protein expression of HIF-α in lung and brain was quantified respectively by compared with GAPDH (**B**, **D**). The values are presented as means ± SD. **P < 0.01 versus control group; ^#^*P* < 0.05 versus ALI group

As displayed in Fig. 7A–D, the Western blot assay illustrated that the HIF-1α level of lung and brain tissues in the ALI group was higher relative to the control group (*P* < 0.01). We further demonstrated that DMF pretreatment reduced the HIF-1α level of lung and brain tissues in LPS-triggered ALI mice (Fig. 6A–D, *P* < 0.05).

## Discussion

The occurrence of ALI is related to many factors. In the ALI animal model, the endotoxin (LPS) infection can simulate clinical lung injury caused by Gram-negative bacillus infection (such as various sepsis) and septic shock caused lung injury [2, 3]. Sepsis and septic shock are prone to occur in patients with poor physical strength after major surgery. In patients undergoing cardiothoracic surgery, ALI is a risk factor that requires close attention during the perioperative period, for it is often associated with poor prognosis and death case of surgery. Hence, exploring new drugs with high efficiency and low toxicity is of great significance for the treatment of ALI.

In LPS-induced ALI, inflammatory cells such as neutrophils are recruited and activated, then infiltrate into the lung tissue and release inflammatory mediators [25]. A variety of inflammatory mediators and cytokines participate in and cause extensive destruction/permeability enhancement of pulmonary microvascular and alveolar epithelium, which in turn lead to the occurrence of ALI [17]. Uchiba et al. found that a large number of neutrophils were aggregated in the lung tissue of rats 30 min after intravenous injection of LPS, and the pulmonary vascular permeability was also evidently enhanced [26]. Abraham et al. found that mice treated with cyclophosphamide or neutrophil antibody apparently improved LPS-triggered pulmonary edema and notably repressed the expression of inflammatory cytokines [27]. Similarly, our research manifested that LPS-induced ALI led to a increase in the numbers of total cells and neutrophils and protein content in BALF, which suggested that LPS could lead to significant inflammation and inflammatory cell infiltration in BALF of ALI. While DMF pre- treatment partially neutralized the effect of LPS. It was suggested that DMF might protect ALI caused by LPS by repressing the aggregation of inflammatory cells to reduce the release of inflammatory mediators and cytokines.

A study confirmed that TNF-α could induce neutrophils to adhere to the vascular endothelium, and promote neutrophils to migrate and penetrate into the bronchoalveolar cavity, causing serious damage to the tissues [28]. The TNF-α induced after LPS stimulation can activate the inflammatory response of the lung, regulate NF-κB transcript various cytokines, induce the aggregation and migration of neutrophils, initiate the inflammatory cascade, and maintain inflammation [29]. NF- κB can be activated by inflammatory cytokines and induce the expression of inflammatory cytokines, which is widely involved in the regulation of inflammatory mediators and pro-inflammatory mediators. Our analysis of ALI-related inflammation-related markers in BALF, serum, and brain tissues showed that LPS caused an increase in the levels of TNF-α, IL-1β, IL-6, as well as the ratios of p-NF-κB/NF-κB and p-IKBα/IKBα, suggesting that LPS may induce lung injury through excessive activation of the NF-κB pathway, which is consistent with the study by Lentsch et al. [30]. Further, we discovered that DMF pretreatment partially offset the promotion of LPS on the levels of TNF-α, IL-1β, IL-6, as well as the ratios of p-NF-κB/NF-κB and p-IKBα/IKBα, suggesting that DMF can inhibit the translocation of NF-κB into the nucleus and reduce the level of inflammation-associated markers, thus alleviating lung tissue damage. The anti-inflammation ability of DMF was also reported in published studies. In thioacetamide-induced liver damage, DMF was proved to exhibit the hepatoprotective potential through the downregulation of inflammatory cascades and upregulation of antioxidant status, including the inhibition of IL-6 and NF-κB [31].

In addition, we performed Morris water maze test on mice to check cognitive function, to complement the lack of research on DMF in ALI-related cognitive impairment. We demonstrated that LPS induced persistent cognitive impairment in ALI mice was partly attenuated by DMF pretreatment, which was reflected in MMM test with decreased escape latency and path length, accompanied with decreased brain water content and BBB partial recovery. Sahu et al. found that olaparib could attenuate ALI-related neurocognitive disorders by restraining inflammatory factors [32]. This suggested that the neuroprotective effect of DME in ALI may be similar. Except for this hypothesis, DMF was also roved to improve cognitive deficits in some studies, including sepsis, ischemic stoke, and Alzheimer’s disease, which indirectly proved the protective effect of DMF on ALI induced cognitive impairment [33–35].

HIF-1α is a transcription factor induced by hypoxia, it could be induced by flammation-related factors in an inflammatory environment [36–38]. Studies have clarified that LPS could induce HIF-1α expression by stimulating HIF-1α aggregation, increasing HIF-1α transcription and protein translation, and inhibiting protein degradation [39, 40], which was consistent with our study. In addition, the hypoxia has important role to the onset of cognitive impairment via the activation of HIF-1α. In mice prolonged exposed to inhaled anesthetics, increases in HIF-1α in the hippocampus was observed, combined with BBB disruption and cognitive dysfunction, while HIF-1α inhibitor YC-1 markedly suppressed the expression of HIF-1α, mitigated the severity of BBB disruption and attenuated cognitive deficits in the MM test [41]. In our study, increased HIF-1α expression was accompanied with cognitive impairment in ALI mice, suggested a direct connection between the two issues. Further, we also found that DMF pretreatment suppressed the HIF-1α level of lung and brain tissues in LPS-triggered ALI mice, as well as the cognitive impairment alleviation.

However, our study also has limitations. Based on these results, we suggested that the protective effects of DMF against lung injury and brain cognitive impairment were associated with the suppression of inflammation, but further in-depth study to confirm and clarify the protective mechanism of DMF on ALI is necessary. Overall, the findings in this research need to be confirmed and validated by larger subsequent research, to pave the way to a therapeutic target in the prevention and management of neurological events in patients with ALI.

In summary, this study fund that DMF has a protective effect against LPS-triggered ALI and cognitive impairment in rat model, and its mechanism may be blocking the activation of NF-κB, reducing the inflammatory cytokines and genes and restraining the inflammatory response.

## Data Availability

All data generated or analyzed and materials used during this study are included in this article.

## Abbreviations

DMF: Dimethyl fumarate
ALI: Acute lung injury
BALF: Broncho-alveolar lavage fluid
BBB: Blood–brain barrier
LPS: Lipopolysaccharide
IL-6: Interleukin-6
TNF-α: Tumor necrosis factor-α
IL-1β: Interleukin-1β
QRT-PCR: Quantitative real-time
p-NF-κB: Phosphorylated-nuclear factor-kappaB
p-IKBα: Phosphorylated-IKBalpha
HIF-α: Hypoxia inducible factor-α
